# New York Letter

**Published:** 1893-07

**Authors:** Wm. L. Russell

**Affiliations:** 151 East 50th Street; New York


### *CORRESPONDENCE.*

### NEW YORK LETTER.

NEW YORK, June 15, 1893.

SYMPHYSEOTOMY.

The revival of this operation has attracted considerable attention here during the past winter. The subject was up for discussion at a recent meeting of the academy. The anatomy of the parts involved was briefly reviewed by Dr. J. E. Kelly. He stated that the symphysis consisted of fibro-cartilage and a synovial space placed between two surfaces of bone which were connected by ligaments. The bony surfaces were often irregular projections fitting into depressions, thus forming a firm connection difficult of division. The symphysis was analogous to the sternum, the degree of anchylosis being simply a matter of development. The muscles which were passed through were the pyramidales and the recti. Between the former was a space bounded posteriorly by the fascia transversalis. It was in this space that the finger was passed downwards to beneath the pelvic arch, the vessels and nerves being pushed backward with the fascia transversalis. The dorsal artery, vein, and nerve of the clitoris were avoided by keeping close to the bone. He thought the incision through the symphysis could best be made by means of a Syme's knife, and that its direction should be from above downward, and from before backward. The separation having been made, the bony masses were enabled to rotate at the sacro-iliac synchondrosis, the antero-posterior diameter of the pelvis was increased, and the removal of the child facilitated. In closing the symphysis, he thought an ordinary staple the best implement with which to secure fixation. Dr. Garrigues, who was the first to perform the operation in this city, said that the history of the procedure dated back to 1768. Its introduction was hailed with enthusiasm, but it was soon abandoned. It was revived in Italy in 1866, and in France in 1891 and 1892. The operation was first done in the United by Dr. Harris of Philadelphia. In a hundred operations previous to 1866, thirty-one women and sixty-five children were lost; in sixty-six operations since

1891, there had been but five deaths in nine countries. There had been but twelve deaths in a hundred and twenty-two cases since 1886, and among the seventeen cases recorded in this country there were but three deaths. The result of the operation was to increase the pelvic diameters considerably. It was useful in cases of tumor, in face presentations with the chin posteriorly, and when there was absolute narrowing of the pelvis with a dead child. The opening was spread by placing the women on her back, with the knees flexed and turned well outwards. He made a long incision in operating, and used a sickle-shaped knife in dividing the symphysis. If the symphysis was ossified or the bones irregular, a drain saw, introduced by means of a long needle might have to be employed. The bladder might be between the child's head and the symphysis, and in such a case should be carefully pushed backwards to avoid wounding. The expulsion of the child could be left to nature, but it was customary to deliver with forceps. The wound should not be closed until the placenta was removed, when deep sutures should be inserted in front of the pubes, and the broad strips of adhesive plaster placed across to support the bones. The patient was to remain in bed for three weeks, with her knees fastened together; being lifted, when necessary, by trochanters.

Since the introduction of this operation, with its modern improvements, it was no longer necessary to wait for the death of the child in many cases of narrow pelvis, and the great mortality of forty-three per cent. of the infants and five per cent. of the women, in cases in which induction of premature labor seemed indicated had been banished. If the antero-posterior diameter were less than two and three-quarters inches, mutilation was still necessary. Many difficult forceps deliveries and versions could, however, be improved upon, the mortality in these cases being very great, when the antero-posterior diameter was less than three and a quarter inches. The operation might also be indicated when the pelvis was normal, but the child's head large. It would soon be known when and how to operate, and the operation would then assume great importance.

Dr. J. C. Edgar said that there could no longer be any doubt of the increase in the pelvic diameters produced by this operation. Experiments had shown that when two and two-fifth inches of separation were effected, the antero-posterior diameter was increased three-quarters of an inch, and the transverse and oblique diameters one inch each. The degree of separation had been even greater also than that mentioned. He had recently done the operation on a case in which the conjugate diameter was not over three and a quarter inches. He had found the head movable above the pelvic brim, and had attempted delivery with the axis-traction forceps. This failing and the uterus being firmly contracted over the child, he decided against any attempt at version, and preferred symphyseotomy to mutilation or Cæsarean section. The patient was prepared as for laparotomy, and an incision was made over the pubes. There was little hemorrhage. The finger was passed down behind the pubes, after pushing the head upwards through the vagina, and an ordinary carved blunt bistoury was passed along the finger. The joint was cut from above downwards and behind forwards, care being taken to depress the urethra by means of a catheter previously placed within it. The head being still movable above the brim, version was done, and the child extracted, though not without difficulty. The child weighed seven pounds, and was nineteen and a half inches long. Silk sutures were passed through the fibrous tissue over the joint, and the parts brought in close apposition. Both mother and child had done well. It was now forty-one days since the operation, and there was good union, the patient being able to move about without difficulty. The sutures were removed on the eighth day, and the patient remained in bed for four weeks. He did not think well of wiring the bones, as satisfactory union resulted without that being done, and it might cause neurosis.

Dr. R. A. Murray said that the operation did not take the place of version, forceps nor Cæsarean section, but occupied its own place in cases in which the pelvic diameters were not too much shortened to prevent success.

Dr. Boldt thought there was great danger of such an operation being done too frequently by over-zealous, practitioners.

Dr. E. H. Grandin said his experience with the operation had led him to conclude that it must be done early and before sepsis had been introduced. If no injury was done to the surrounding tissues, no disability resulted. It saved the child in minor degrees of contraction, and reduced the limit for Cæsarean section and destructive operations to two and three quarter inches for the conjugate diameter. It was better than protracted traction with the forceps in impacted occipito-posterior cases. Accurate pelvimetry was essential. He thought the long primary incision unnecessary, and it increased the danger of hemorrhage and of septic infection. When the finger was hooked under the symphysis, the field was controlled. A glass catheter should be inserted in the urethra, and the vesical neck depressed; in this way the danger of causing vesical fistula was avoided. Careful asepsis was essential to success. The incision should be made downward and inward through the symphysis by a blunt pointed bistoury. He did not believe wiring, nor very close coaptation to be necessary. He believed that the operation would lessen the sacrifices of children and the number of idiots and epileptics produced by difficult labor.

Three cases were shown at the meeting, illustrating the absence of disability after the operation. They all walked, ran, hopped, and walked up-stairs without difficulty.

### EXPERT TESTIMONY.

Two recent murder trials here, in which expert medical testimony has been a prominent feature, have attracted considerable attention to that subject. There is a general impression that reform in the methods of providing such testimony is essential. To the ordinary observer, however, reform of those who pose as experts seems to be the essential thing. It is hard to know how to account otherwise for a recent written opinion by a number of our most prominent neurologists to the effect that a juryman who was taken sick during a trial, and whom not one of them had examined or even seen, had epilepsy, the opinion being made the basis of a demand for a new trial of a man convicted of murder. It is mortifying to have to record the fact of scientific men exposing themselves to the well-deserved rebuke which they received at the hands of the judge and district attorney.

At a recent meeting of the Society of Medical Jurisprudence, Dr. E. C. Spitzka read a paper entitled "Recent Forensic Malpractice," in which he characterized the present methods of obtaining expert medical testimony as calculated to keep qualified men off the stand, and as tending to unnecessarily prolong a trial and to confuse a jury. He believed that the medical examination in criminal cases should be controlled by both sides.

Dr. White, of Brooklyn, seemed to be satisfied with present methods, and stated that no man really qualified as an expert need be afraid of any cross-examination by a lawyer.

Mr. Henry Morrison, a lawyer, thought it a question if the expert testimony should not be submitted to those selected by the judge to be associated with him in judging of its proper significance and value.

Dr. M. D. Field thought the difficulty lay in the fact that opposing lawyers were not seeking the truth, and that experts usually set out to help the side employing them as much as possible.

A lawyer expressed himself of the opinion that there was a growing tendency to produce dramatic and sensational effects. This had become notoriously true of the newspapers, and it had also crept into the courts. The lawyer of the one side was too ready with a sneer for the witness of the other, an eminent scientist appearing in a recent trial having been referred to as a ptomaine crank. The control of expert testimony seemed to depend largely on the length of one's purse. It might be a good plan for the district attorney to have a medical assistant to investigate the medical facts of each case. The number of experts should then be limited, and should be selected by the court.

Dr. B. Sachs said that it should be the aim of any physician called as an expert to determine absolute truth and simple justice without reference to the attitude of the lawyer employing him. It would be better if the expert even were selected by the court, and because he is, in fact, an expert on the subject under consideration. It was the lawyer's business to work for the interests of his client, and absolute truth and justice were not his aims. Really scientific men were at present inclined to withdraw from expert practice. They were obliged to submit their evidence hurriedly, and from facts imperfectly presented to them, and did not receive the respect due them from opposing lawyers.

The general sentiment of the meeting seemed to be in favor of a change in the present methods, and a committee was appointed to take some action in the matter.

One especially good point brought out was in reference to post-mortem examinations and chemical analyses, which are usually made entirely under direction of the prosecution, the defence being without representation.

WM. L. RUSSELL.

151 *East 50th Street.*

